# Draft Genome Sequence of a Hexachlorocyclohexane-Degrading Bacterium, *Sphingobium baderi* Strain LL03^T^

**DOI:** 10.1128/genomeA.00751-13

**Published:** 2013-09-19

**Authors:** Jasvinder Kaur, Helianthous Verma, Charu Tripathi, J. P. Khurana, Rup Lal

**Affiliations:** Molecular Biology Laboratory, Department of Zoology, University of Delhi, Delhi, Indiaa; Interdisciplinary Centre for Plant Genomics & Department of Plant Molecular Biology, University of Delhi South Campus, New Delhi, Indiab

## Abstract

*Sphingobium baderi* strain LL03^T^ was isolated from hexachlorocyclohexane (HCH)-contaminated soil from Spolana, Czech Republic. Strain LL03^T^ is a mutant that is deficient in *linB* and *linC* (genes that encode hexachlorocyclohexane haloalkane dehalogenase and dehydrogenase, respectively). The draft genome sequence of LL03^T^ (~4.85 Mb) consists of 92 contigs and 4,914 coding sequences, with a G+C content of 63.5%.

## GENOME ANNOUNCEMENT

The unregulated disposal of hexachlorocyclohexane (HCH) muck (consisting of α- and β-HCH) has resulted in the creation of a large number of HCH dumpsites around the world during the last 50 years (1). Interestingly, several sphingomonads that degrade HCH isomers have been isolated from these HCH dumpsites from different geographical locations (2–4). Earlier, we reported the genome sequence of sphingomonads isolated from the HCH dumpsite in India (5–8). Here, we report the genome sequence of *Sphingobium baderi* strain LL03^T^, which was isolated from the HCH dumpsite situated in Spolana, a former Czech producer of lindane (9).

The genomic DNA of LL03^T^ was sequenced by using 454 GS FLX Titanium and Illumina genome analyzer platforms. The sequencing data of ~514 Mbp were assembled into 92 contigs by using the ABySS 1.3.3 assembler (47 k-mer) (10). The final assembly (*N*_50_ contigs, 269 kb) was validated based on the paired-end information. Annotation was done using RAST version 4.0 (11) and NCBI Prokaryotic Genomes Automatic Annotation Pipeline (PGAAP) version 2.0 (http://www.ncbi.nlm.nih.gov/genomes/static/Pipeline.html).

The draft genome sequence (~4.85 Mb) has a coding density of 88.95%, with a GC content of 63.5%. A total of four rRNA operons (5S-16S-23S) were identified by using RNAmmer (12). Also, a confirmed clustered regularly interspaced short palindromic repeat (CRISPR) with 22 spacers was identified in the draft genome (13). Genes for phenol, anthranilate, and homogentisate degradation were also found in the assembled contigs. Such gene clusters have already been reported from genomes of sphingomonads isolated from the HCH dumpsite in India (5, 6, 8).

We have already reported the absence of *linB* from the genome of *Sphingobium quisquiliarum* P25^T^, isolated from the HCH dumpsite in India (8). In the draft genome sequence of *S. baderi* LL03^T^, both *linB* and *linC* encoding HCH haloalkane dehalogenase and dehydrogenase, respectively, were found to be absent from all of the *lin* genes (*linA* through *linF*) that are required for the degradation of HCH isomers (1). This strain was also found to contain 22 copies of IS*6100* elements that are known for their active roles in the horizontal transfer of *lin* genes (14). Based on these observations, we hypothesized that strain LL03^T^ is yet to acquire *linB* and *linC*, probably through horizontal gene transfer (HGT) (15).

Discerning the HCH degradation pathway of HCH isomers in this strain, especially with highlighting of the absence of *linB* and *linC*, will be of much interest (1). Additionally, the genome of strain LL03^T^ can be a potential source of information, to be studied along with the available metagenomic data and genomes of other HCH-degrading sphingomonads (5–8, 15, 16), for understanding the details involved in HCH degradation (17) and the mechanisms for the acquisition of *lin* genes.

### Nucleotide sequence accession numbers.

The draft genome sequence of *Sphingobium baderi* LL03^T^ is available in GenBank under the accession number ATIB00000000. The version described in this paper is the first version, ATIB01000000.
